# Rethinking Diabetes from the Perspective of Diverse Insulin Actions in Various Organs

**DOI:** 10.31662/jmaj.2024-0063

**Published:** 2024-10-03

**Authors:** Kohjiro Ueki

**Affiliations:** 1 Department of Molecular Diabetic Medicine, Diabetes Research Center, Research Institute, National Center for Global Health and Medicine, Tokyo, Japan; 2 Department of Molecular Diabetology, Graduate School of Medicine, the University of Tokyo, Tokyo, Japan

**Keywords:** Diabetes, Insulin action, Insulin secretion, Cancer, Sarcopenia, MASH, Microbiome

## Abstract

Diabetes mellitus is defined as a group of metabolic diseases characterized by chronic hyperglycemia based on insufficient insulin action. At present, treatment for diabetes aims to prevent micro- and macrovascular complications. Although advances have been made in methods of controlling the risk factors of complications, including blood glucose management, there is still no effective treatment to cure diabetes. This is largely because we do not fully understand what diabetes is. To cure diabetes, it is necessary to elucidate the whole picture of insulin actions including those other than metabolic actions in various tissues and to understand what disorders are caused by its reduction or excess. This article reviews diverse insulin actions in various organs and the effects of their deficiency on diabetes, its complications, and associated diseases.

## What Is Diabetes?

Diabetes mellitus is defined as a group of metabolic diseases characterized by chronic hyperglycemia caused by insufficient insulin action ^(1)^. Diabetes causes neuropathy, retinopathy, and nephropathy specific to diabetes and increases the risk of atherosclerotic diseases such as myocardial infarction and stroke, resulting in shortened lifespan and reduced quality of life. Currently, the blood glucose and HbA1c thresholds used as diagnostic criteria for diabetes are set at values that increase the prevalence of diabetic retinopathy. In terms of treatment, the major goal is to prevent macroangiopathy in addition to microangiopathy, which has a significant impact on the choice of drug therapy in guidelines. This conception of diabetes as a disease and its therapeutic goals may be problematic. Despite the wide range of actions of insulin, is it appropriate to define the disease as characterized only by abnormal glucose metabolism, is it appropriate to set the suppression of complications as a therapeutic goal, or is it not possible to cure diabetes mellitus by normalizing insulin actions?

## Insulin Action in the Development of Diabetes, Its Complications, and Diabetes-associated Diseases

**Figure 1.**
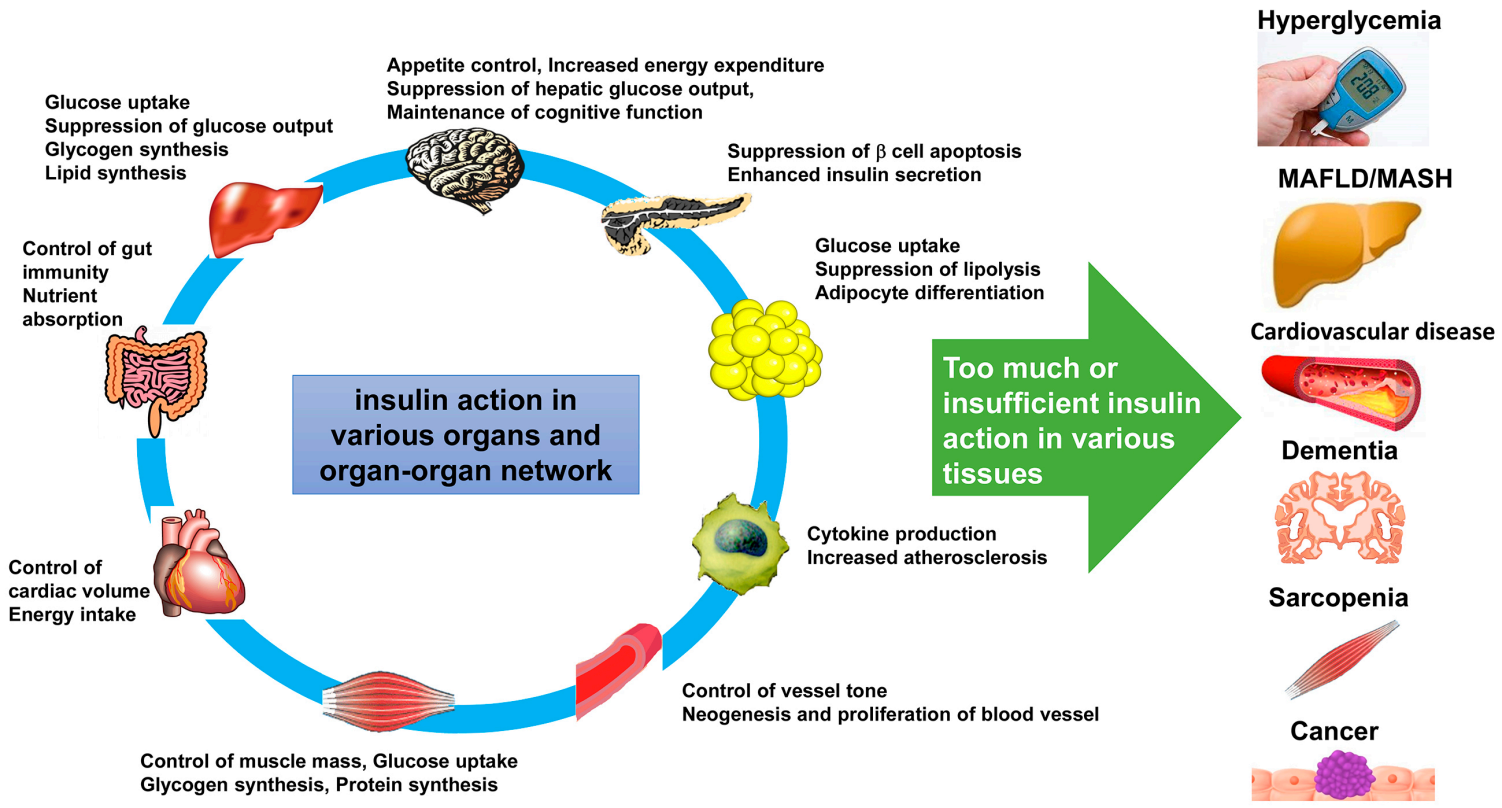
Various insulin actions and diseases caused by insufficient or excess of insulin actions.

Insulin binds to its receptor, a tyrosine kinase, and phosphorylates insulin receptor substrate (IRS) proteins, thereby activating PI3 kinase (phosphoinositide-3 kinase) and its downstream molecules, such as Akt, which is responsible for metabolic and antiapoptotic effects, while it also activates Erk pathway that is mainly responsible for proliferative effects ^(2)^. In diabetes, insulin signaling in the liver, skeletal muscle, and adipocytes is impaired, resulting in abnormal glucose metabolism. however, insulin receptors are expressed in almost all organs and have been shown to have various functions other than metabolic action (Figure 1). In lower animals such as *Caenorhabditis elegans* and flies, inhibition of insulin signaling has been shown to suppress aging and extend lifespan, but in mammals including humans, diabetes, a condition in which insulin action is impaired in metabolism, increases the risk of aging-related diseases such as atherosclerosis, cancer, dementia, and sarcopenia, and if the condition is not adequately controlled, life expectancy is also thought to be shortened ^(3)^.

In diabetes, reduced insulin action in the liver, skeletal muscle, and adipocytes causes metabolic disorders, in which case other nonmetabolic actions in other organs may be expected to be impaired as well. Conversely, hyperinsulinemia is often observed in the early stages of type 2 diabetes to compensate for the decreased metabolic effects of insulin, and indeed, this hyperinsulinemia may enhance certain insulin actions. By analogy with findings in lower animals, some of the diabetic complications and diseases highly associated with diabetes have been thought to be caused by excessive insulin action. In fact, hyperinsulinemia is associated with cardiovascular disease ^(4)^ and colorectal cancer ^(5)^. We cannot overcome diabetes until we understand why this difference occurs between mammals and the lower animals and which organs in diabetes are impaired by insufficient or excessive insulin action, causing complications and diabetes-associated diseases (Figure 1), which we have been trying to explore.

## Insulin Action in the Pancreatic β Cells

Type 2 diabetes consists of two pathological conditions: insulin resistance, which is mainly caused by environmental factors such as obesity, and a decrease in the quantity and function of β cells, which is greatly influenced by genetic predisposition as suggested by recent genome-wide analysis, but the quantity and function of β cells decrease over time, and diabetes is said to develop and progress ^(1), (2), (6), (7)^. However, when hyperglycemia is corrected by the glucose-lowering agents, such as insulin therapy, insulin secretion is restored, a phenomenon known as release of “glucose toxicity,” and correction of insulin resistance factors such as obesity also improves insulin secretion. This suggests that some acquired factors also affect the quantity and function of β cells. It has been generally understood that these phenomena reduce the load on β cells by decreasing the insulin requirement, thereby restoring temporarily impaired β cell function.

Pancreatic β cell-specific insulin receptor knockout mice (βIRKO mice) developed by Kahn’s group show impaired glucose-stimulated insulin secretion, especially in the first phase, although there is no significant decrease in β cell mass ^(8)^. The βIRKO mice have worsening glucose tolerance over time despite the absence of insulin resistance in peripheral tissues, suggesting that impaired insulin action in β cells is involved in the development of type 2 diabetes mellitus. Furthermore, β cell-specific knockout mice for the IGF-1 (insulin like growth factor-1) receptor, which shares a downstream signaling molecule with the insulin receptor, also show a phenotype similar to that of βIRKO mice ^(9)^. In these knockout mice, when one receptor is knocked out, the signal from the other receptor may work compensatory to weaken the phenotype, so when β cell-specific insulin and IGF-1 receptor double knockout mice (βDKO mice) are created, these mice develop marked hyperglycemia and all die within 7 weeks of age due to severe diabetes. βDKO mice, unlike the single knockout mice, show a marked decrease in β cell mass due to increased apoptosis ^(10)^. In addition, the homodeficiency of the IGF-1 receptors with heterodeficiency of the insulin receptors results in mild hyperglycemia and decreased β cell mass, while the homodeficiency of the insulin receptors with heterodeficiency of the IGF-1 receptors results in a phenotype similar to that of βDKO mice, suggesting that the autocrine/paracrine insulin is important for the regulation of pancreatic β cell volume.

By what mechanism does insulin regulate the amount and function of pancreatic β cells? Downstream of the insulin receptor, glucose-stimulated insulin secretion is impaired in islets of mice lacking its substrate, insulin receptor substrate-1 (IRS-1), while pancreatic β cell volume is maintained ^(11), (12)^. Nevertheless, mice deficient in IRS-2 develop overt diabetes due to decreased pancreatic β cell mass ^(13), (14)^, suggesting that IRS-2-mediated signaling may be the major signaling pathway for the regulation of pancreatic β cell volume ^(15), (16)^. We have generated pancreatic β cell-specific knockout mice of the p85 subunits of PI3K, one of the two major components that transmit signals downstream of the insulin receptor ^(17)^. These mice exhibit impaired glucose tolerance due to decreased glucose-stimulated insulin secretion. This is thought to be due to decreased expression of SNARE proteins required for fusion and release of insulin granules to the plasma membrane and of the component proteins of the gap junction, which regulates synchronous insulin secretion through β cell communication. However, despite increased apoptosis, the decrease in β cell mass is only mild, suggesting that the loss of PI3K abolishes negative feedback to MEK/Erk signaling, resulting in increased Erk activity, which in turn increases cell proliferation. Therefore, we have generated pancreatic β cell-specific MEK knockout mice, which show impaired glucose tolerance due to decreased β cell mass compared to the control mice, when they are on a high-fat diet ^(18)^. This is due to decreased cell proliferation, although apoptosis is not increased. On the other hand, glucose-stimulated insulin secretion is also decreased in isolated islets, possibly because the insulin secretory mechanism is also impaired due to decreased phosphorylation of SNARE proteins and cytoskeletal proteins.

**Figure 2.**
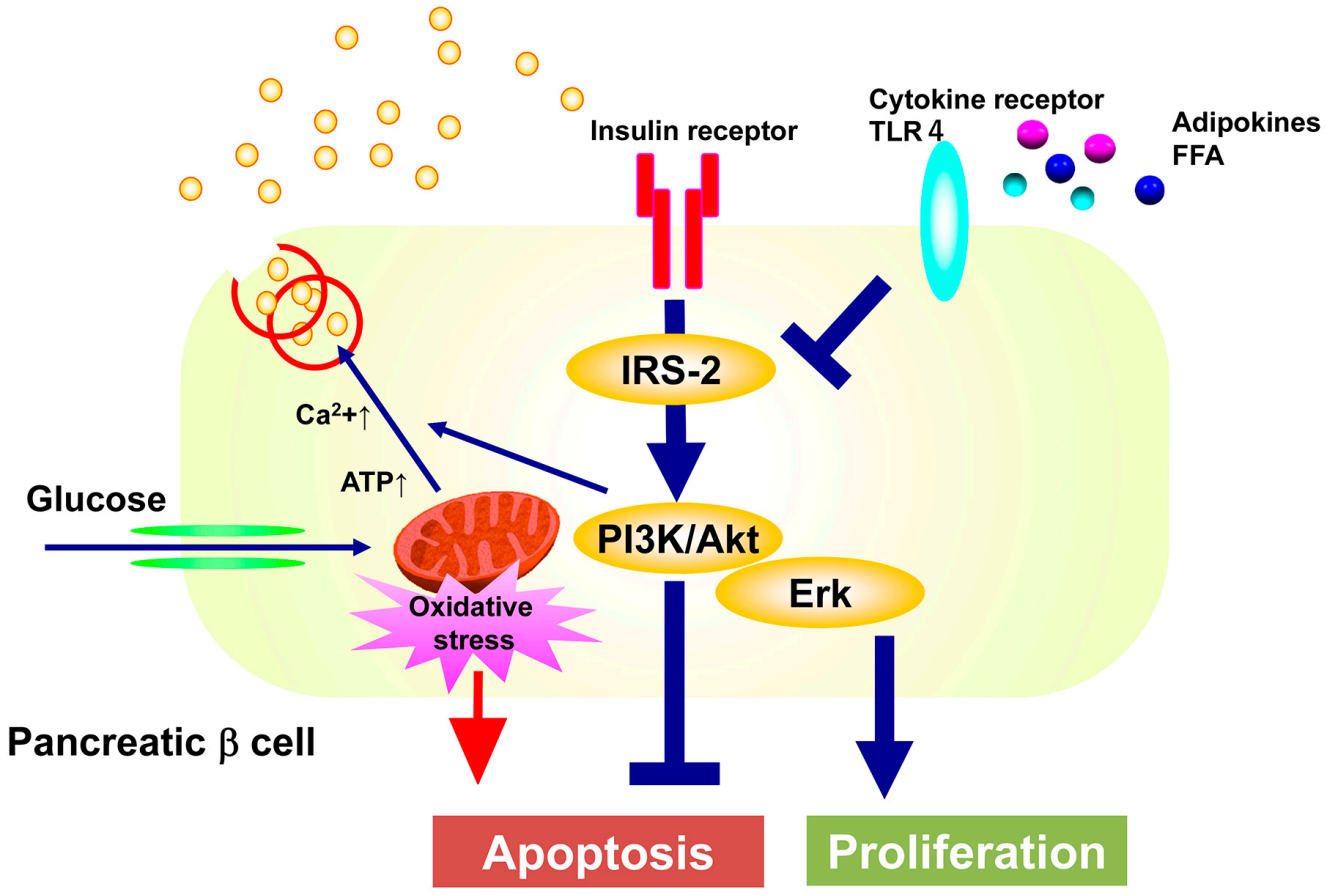
Roles of insulin action in the regulation of pancreatic β cell function and mass.

**Figure 3.**
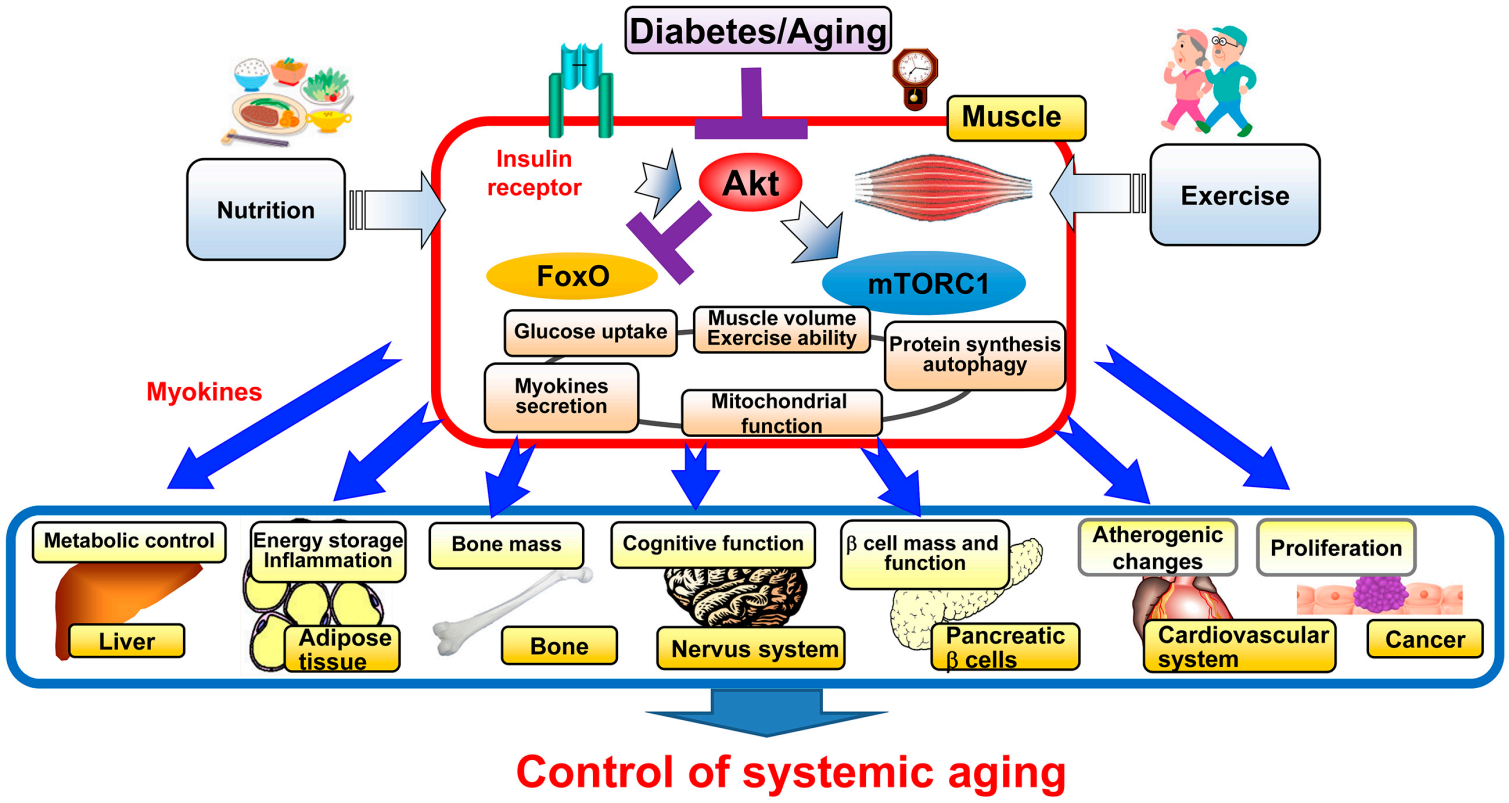
Roles of insulin action in the regulation of systemic aging through controlling skeletal muscle quantity and quality Insulin action controls muscle volume by regulating mitochondrial quantity and quality in the skeletal muscle. In diabetes, impaired insulin action in the skeletal muscle leads to sarcopenia and presumably alters production of myokines, which promote aging-related disease and systemic aging.

Taken together, the above findings suggest the following mechanisms for the maintenance of normal pancreatic β cell quantity and function. Pancreatic β cells are extremely vulnerable to oxidative stress caused by mitochondrial reactive oxygen species generated during glucose metabolism and are prone to apoptosis. However, insulin secreted in response to glucose binds to its receptors in an autocrine and paracrine manner and activates the PI3K pathway to inhibit apoptosis and the MEK/Erk pathway to promote cell proliferation to maintain or increase pancreatic β cell volume as needed. Both pathways also regulate the expression and activity of factors that control the secretory process of insulin to maintain normal insulin secretory capacity (Figure 2). In diabetes, the input of insulin signals to pancreatic β cells is decreased due to decreased insulin secretion caused by genetic predisposition, and the insulin signaling of pancreatic β cells is further impaired by lipids, adipokines, etc., resulting in decreased PI3K and MEK/Erk activity, increased apoptosis of pancreatic β cells, and impaired proliferation, resulting in decreased pancreatic β cell volume. This may lead to a vicious cycle of decreased insulin secretion and decreased insulin signaling in pancreatic β cells (Figure 3).

This concept is the first to show that the two major pathogeneses of type 2 diabetes, insulin resistance and insulin insufficiency, which were previously thought to be completely separate, interact with each other and have become a theoretical pillar of current diabetes treatment, as lowering blood glucose levels and reducing glucose influx into pancreatic β cells can preserve β cell volume and function, thereby improving the long-term prognosis of diabetes. In addition, treatment to enhance insulin action in pancreatic β cells may also be effective, but it is unclear how much direct effect existing insulin sensitizers have on pancreatic β cells. A molecule called inceptor, which removes insulin and IGF-1 receptors from the cell membrane surface of pancreatic β cells and reduces insulin and IGF-1 signaling, has recently been identified ^(19)^. Inhibition of inceptor may be a new diabetes treatment that enhances insulin action in pancreatic β cells, and the development of inhibitors is expected.

## Decreased Insulin Action in the Development of Sarcopenia

Currently, more than 70% of people with diabetes in Japan are 65 years old or older, and prevention and treatment of geriatric syndromes such as sarcopenia, frailty, and dementia is an important issue since the risk of these syndromes is increased by diabetes and is an aggravating factor of diabetes ^(20)^. We have been studying the effects of diabetes on the aging process in diabetic mice and found that the activity of Akt, a key molecule for insulin signaling, is decreased in diabetic and aging mice, resulting in loss of muscle mass mainly in fast-twitch muscles, similar to sarcopenia in humans. Indeed, skeletal muscle-specific knockout of Akt1 and Akt2, major isoforms of Akt, results in aging phenotypes such as premature sarcopenia and bone loss with reduced mitochondrial mass and functions, as well as a shortened lifespan. When these mice are subjected to a high-fat diet to produce sarcopenic obesity, they show a shortened lifespan due to increased cancer and the possibility that some factor (myokine) secreted by the sarcopenic skeletal muscle may promote cancer growth. Such sarcopenia and shortened lifespan are ameliorated by suppression of the transcription factor FoxO ^(21)^. Therefore, it is promising that enhancement of insulin action in the skeletal muscle, especially suppression of FoxO, is effective in preventing sarcopenia in diabetes and that myokines from the skeletal muscle can be identified and targeted for prevention and treatment of aging-related diseases (Figure 3).

## Insulin Action and MASH and Its Associated Liver Cancer

Cancer is currently the leading cause of death among Japanese people with diabetes, with liver cancer ranking second following lung cancer. The risk of developing hepatocellular carcinoma is approximately double in the people with diabetes, and prevention of hepatocellular carcinoma is important for improving the prognosis of these people. In recent years, due in part to advances in antiviral therapy, the incidence of hepatocellular carcinoma due to hepatitis C has decreased, and the incidence of liver cancer due to metabolic dysfunction-associated steatotic liver disease (MASLD) or metabolic dysfunction-associated steatohepatitis (MASH) has increased to more than 40% of the total cases and the large portion of those are affected by diabetes ^(22), (23)^. Most previous studies of MASH and its associated hepatocellular carcinoma have focused on clinical studies in obese Western people or in animals with severe obesity or fed on special diets, with the idea that excessive insulin action due to hyperinsulinemia is involved in the pathogenesis of the disease. We have been studying the mechanism of MASH and its associated hepatocarcinogenesis in people with diabetes in Japan and east Asians, who are mainly nonobese or mildly obese.

**Figure 4.**
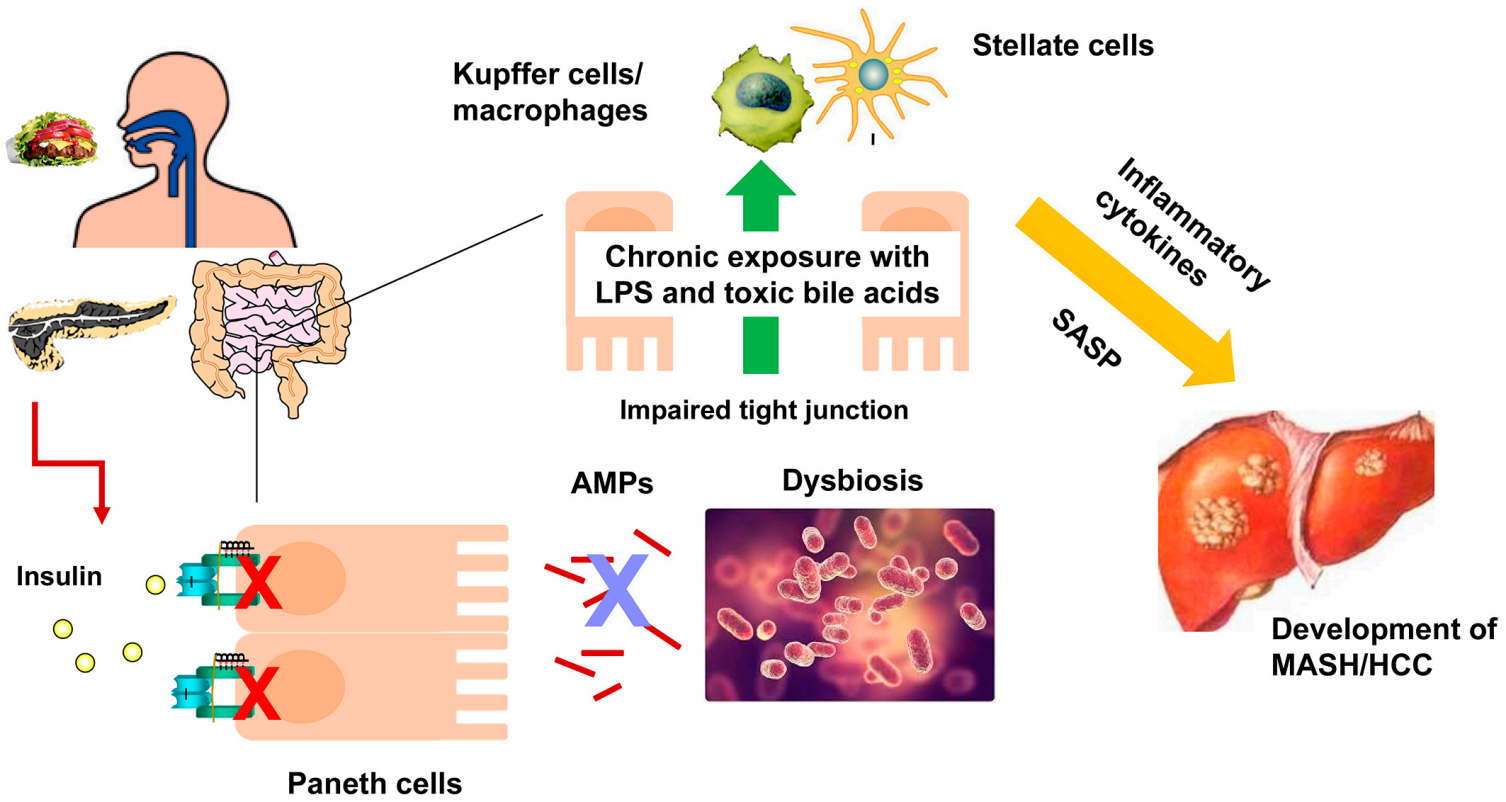
Roles of insulin action in the prevention of MASH and its related liver cancer through controlling gut barrier function In diabetes, production of antimicrobial peptides (AMPs) regulated by insulin action in Paneth cells is impaired, leading to dysbiosis and subsequent chronic exposure of LPS and toxic bile acids to Kupffer and stellate cells. These cells then secrete inflammatory cytokines and senescence-associated secretory phenotype, which promote carcinogenesis in the liver.

More than 60% of the origin of fat accumulation in the liver is thought to be an influx of free fatty acids due to lipolysis of adipocytes, which are increased in diabetes due to impaired insulin action in adipocytes. The expression of peroxisome proliferator-activated receptor γ (PPARγ) in the liver is upregulated in the diabetic state and exacerbates the influx and accumulation of fatty acids. We have found the antisense RNA encoded in the IRS2 locus in mice and humans, which is upregulated in feeding, obesity, and diabetes and upregulates PPARγ expression, leading to increased hepatic fat accumulation ^(24)^. Furthermore, feeding induces endoplasmic reticulum (ER) stress in the liver, which is transiently terminated by the induction of a molecule regulating ER-associated degradation, Sdf2l1, when insulin function is normal, but not in diabetes, resulting in prolonged ER stress, exacerbation of diabetes, and development of MASH ^(25)^. In addition, diabetes is associated with impaired intestinal barrier function resulting in dysbiosis ^(26), (27)^. We found that in diabetes, the antimicrobial peptides secreted from Paneth cells in the small intestine are reduced due to decreased insulin action in the intestinal tract, and the intestinal microbiota is altered, increasing the production of toxic secondary bile acids and inducing hepatocarcinogenesis (Figure 4) ^(27)^. These results suggest that suppression of antisense IRS2, suppression of ER stress, and enhancement of intestinal insulin action may be effective in preventing MASH and its hepatocellular carcinoma in diabetes.

## Toward a World without Diabetes

As described above, it is necessary to rethink the disease concept and diagnostic criteria for what we currently call “diabetes mellitus” to include various disorders caused by insufficient or excessive insulin action in addition to abnormal glucose metabolism. Based on such a concept, understanding which insulin actions are deficient and which are excessive in individuals with diabetes will lead to the optimization of treatment.

## Article Information

This article is based on the study, which received the Medical Award of The Japan Medical Association in 2023.

### Conflicts of Interest

None

### Acknowledgement

This work has been supported by Grants-in-Aid for Scientific Research (B) (21390278) and (S) (15H05789) and a Grant-in-Aid for Challenging Exploratory Research (21659227) from the Ministry of Education, Culture, Sports, Science and Technology of Japan (MEXT) and grants for the Research Program on Hepatitis from Japan Agency for Medical Research and Development (JP17fk0210304, JP18fk0210040, JP19fk0210040, JP20fk0210040, JP21fk0210090).

### Disclaimer

Kohjiro Ueki is one of the Editors of JMA Journal and on the journal’s Editorial Staff. He was not involved in the editorial evaluation or decision to accept this article for publication at all.
